# Influence of Awe on Green Consumption: The Mediating Effect of Psychological Ownership

**DOI:** 10.3389/fpsyg.2019.02484

**Published:** 2019-11-07

**Authors:** Liying Wang, Guangling Zhang, Pengfei Shi, Xingming Lu, Fengsen Song

**Affiliations:** ^1^School of Economics and Management, Wuhan University, Wuhan, China; ^2^All China Federation of Supply and Marketing Cooperatives, Beijing, China; ^3^Antai College of Economics and Management, Shanghai Jiao Tong University, Shanghai, China

**Keywords:** awe, psychological ownership, green consumption, interdependent self-construal, independent self-construal

## Abstract

The increasing demand for environmental protection has given rise to burgeoning research on green consumption. The present research adds to this expanding literature by investigating a novel predictor of consumer green consumption: awe. As a self-transcendent emotion, awe arises when people encounter perceptually vast stimuli that overwhelm their existing knowledge and mental structures, and, meanwhile, this also elicits a need for accommodation. This research proposes and demonstrates that, compared with happiness and a neutral affective state, experience of awe promotes green consumption via an enhanced psychological ownership of nature. Moreover, this research identifies a condition by showing that, while awe promotes green consumption when interdependent self-construal is activated, this effect diminishes when independent self-construal is activated.

## Introduction

Environmental degradation and global warming have caused adverse health, economic, environmental, and social impacts on a national level as well as on a global scale. It has been shown that these consequences are largely due to people’s consumption behaviors (Chan, 2001; Jorgenson, 2003). Green consumption has therefore been increasingly and actively advocated for by government policymakers and opinion leaders from various fields to address the negative impacts of environmental degradation (Sheth et al., 2011; Karmarkar and Bollinger, 2015). At the same time, a lot of research has been done from diverse perspectives in the field of sustainability, all pointing to the importance of green consumption (Chan, 2001; Jorgenson, 2003; Lee, 2008). Here, green consumption is defined as consumption behavior aimed at conserving resources and protecting the environment (Carlson et al., 1993).

Despite these research efforts, outcomes are less than ideal (Gleim et al., 2013; Shaw et al., 2016). One key reason is that the majority of green consumption research has been conducted from a constraint perspective, applying an analytical framework of anthropocentric development (Thompson and Barton, 1994; Cheung et al., 2014; Shaw et al., 2016) but ignoring unique characteristics of green consumption and people’s desire for autonomous choice (Moeller et al., 2011; Karmarkar and Bollinger, 2015; Kouchaki et al., 2018). In reality, the belief that human beings are dominant over nature does not provide a basis for choosing to implement sufficient changes that favor green consumption, which is based on altruistic motives for others’ and nature’s sake (Thompson and Barton, 1994; Milfont and Sibley, 2014). Thus, changing the perceived relationship between humans and nature is of vital importance for facilitating green consumption. In this research, we propose a novel way of achieving such change: feelings of awe.

Awe arises when people encounter something vast that is beyond their current knowledge and existing mental structures, provoking a need to update their mental schemas (Keltner and Haidt, 2003). Awe is a self-transcendent emotion that enables individuals to be less concerned about immediate benefits and costs, to resist short-term temptations and desires, and to pay greater attention to the welfare of others and the environment (Piff et al., 2015; Jiang et al., 2018). Awe has attracted considerable attention from researchers in the fields of theology, philosophy, sociology, and psychology, whereas this attention is limited and insufficient in marketing. Research on the role of awe in marketing is still in the primary stage, with only a few recent studies examining the ideas that awe promotes experience innovation (Rudd et al., 2018) and reduces variety-seeking behavior (Cao and Wang, 2018). For instance, Piff et al. (2015) and Zhao et al. (2018) suggest that individuals with a stronger experience of awe are more likely to engage in pro-social and pro-environment behaviors (Griskevicius et al., 2010; Karmarkar and Bollinger, 2015). Thus, it is necessary to explore how green consumption is influenced by awe. Accordingly, in order to extend research on this topic, this paper examines awe in the context of green consumption and attempts to answer the following questions:

Q1: How does awe affect green consumption?
Q2: What is the psychological mechanism by which awe affects green consumption?

We also attempt to identify a condition under which the facilitative effect of awe on green consumption turns off.

To solve the above problems, we propose that awe is an important driver for consumers to participate in green consumption. Through a survey of 589 respondents and four experiments, these experimental results are used to provide evidence of the underlying mechanism and demonstrate that the effect of awe on green purchase behavior is driven by psychological ownership. Besides, after the experience of awe, participants with a tendency toward interdependent self-construal showed higher psychological ownership of nature and possibility of participating in green consumption, while there was no significant difference among participants who had independent self-construal.

This paper contributes to the literature in several ways. First, while most studies on green consumption have been conducted using a constraint perspective, this study takes the perspective of a positive emotion, awe. The objective of green development, namely the achievement of a resource-efficient and environmentally friendly society, only results from everyone’s participation in green consumption. Unfortunately, however, at present only a small proportion of individuals actively participate in green consumption, and those who advocate green consumption tend to exhibit inconsistent behavior when actually buying the relevant products (Gleim et al., 2013; Shaw et al., 2016). Positive emotional experiences have been proven to satisfy the autonomous needs of individuals (Bagozzi et al., 1999; Kouchaki et al., 2018); therefore, it is possible that such experiences may be effective in promoting green consumption. In the present study, we construct a green consumption model using awe as the independent variable with the aim of broadening participation in and embracement of green consumption.

Second, although many researchers have analyzed the psychological mechanism underlying the participation in green consumption, they have found that the subjective initiative of individuals is weak. Pierce et al. (2003) suggest that individuals have a stronger sense of protection and responsibility with respect to their own items or perceived possessions than toward those of others. In the present study we therefore prove that the psychological ownership of nature is a mediator that can foster in individuals the perception that environmental protection is their own responsibility, which in turn better mobilizes their subjective initiative and enthusiasm for participation.

Third, this research identifies an important moderator of the effect of awe on green consumption: self-construal. Specifically, we found that, while awe boosts green consumption when interdependent self-construal becomes salient, this effect diminishes when independent self-construal is activated. Consumer psychology and behavior literature have found significant differences between independent and interdependent self-construal (Singelis, 1994; Wu et al., 2018; Lee and Pounders, 2019). Self-construal theory indicates that individuals with interdependent (vs. independent) self-construal attach more importance to the connection between the self and larger entities (Wu et al., 2018), including nature. Moreover, the stronger the individual’s psychological ownership, the stronger their connection with the target will be (Pierce et al., 2003; Van Dyne and Pierce, 2004). It is therefore necessary to explore the moderating effect of self-construal.

## Awe and Green Consumption

Awe is a unique and powerful emotion (Keltner and Haidt, 2003; Piff et al., 2015) that comprises three properties: (1) it conveys perceptual vastness – that is, the perception that something is immense in size, quantity, scope, complexity, ability, or social bearing (e.g., fame, authority) (Keltner and Haidt, 2003; Piff et al., 2015); (2) it conveys a need for accommodation – that is, the expansion of one’s general reference frame and the formation of a new cognitive structure in response to stimuli beyond one’s existing understanding of the world (Keltner and Haidt, 2003; Piff et al., 2015); and (3) it entails a thirst for innovation – that is, the increasing desire to explore new knowledge and experiences owing to frustration and loss of control when existing knowledge and psychological structure are challenged (Keltner and Haidt, 2003; Piff et al., 2015; Rudd et al., 2018). The sense of time abundance created by focusing on the present also increases people’s motivation to experience innovation (Keltner and Haidt, 2003; Rudd et al., 2012, 2018; Piff et al., 2015). Previous studies have shown that these specific functions of awe have significant effects on individual behaviors, such as an increasing willingness to help others, to donate, to participate in collective behavior, and to adopt pro-environment attitudes (Keltner and Haidt, 2003; Piff et al., 2015; Prade and Saroglou, 2016; Bai et al., 2017; Stellar et al., 2017; Jiang et al., 2018; Zhao et al., 2018). In response to the increasing urgency for green development and Rudd et al. (2018) call to advance research on awe, we extend the concept of awe to the current context of green consumption and posit that it will promote related green development behaviors; this is for several reasons.

First, awe encourages individuals to accommodate the needs of natural development and act in a way that benefits others or larger entities (Keltner and Haidt, 2003; Piff et al., 2015). As environmental problems become increasingly serious, the harmonious development of the human-nature relationship has become more strongly challenged. The accommodation function of awe holds that we should reexamine our attitude toward nature, correct existing unhealthy lifestyles and consumption modes, and develop a new cognitive system of harmonious coexistence between humans and nature (Keltner and Haidt, 2003; Cheung et al., 2014; Rudd et al., 2018; Zhao et al., 2018).

Second, faced with the vastness of nature, people who experience awe become less self-centered and more considerate of others and the broader external environment (Keltner and Haidt, 2003; Piff et al., 2015). The values of self-transcendence reflected by awe serve as the internal impetus to stimulate actions that prioritize environmental protection and are powerful determinants of pro-social behavior (Keltner and Haidt, 2003; Piff et al., 2015; Zhao et al., 2018). As a typical pro-social and pro-environment behavior, green consumption is highly likely to be conducted under the influence of an awe experience.

Third, awe inspires people to embrace new information and experiences openly. Rudd et al. (2018) conducted a series of experiments and found that awe increases people’s need to undergo experiential creation. Broaden-and-build theory also holds that positive emotions such as awe foster the individual’s desire to innovate, explore novel lifestyles, and accept new information and experiences (Fredrickson, 2001) as awe comprises the elements of wonder and curiosity (Haidt, 2003; Rudd et al., 2018). Individuals who have undergone a strong awe experience are more likely to engage in green consumption; this is because green consumption is a new consumption experience that reflects the value orientation of fashion and conforms to the trend of development (Lao, 2013). We therefore posit the following:

H1: Awe is positively associated with green consumption.

## The Mediating Effect of Psychological Ownership

Psychological ownership is the psychological state of an individual that considers the target of ownership, or at least part of that target, as “mine” or “ours” (Pierce et al., 2003). Such a state emphasizes the psychological, rather than the legal, sense of possession and reflects on “the psychological integration of object and me” (Pierce et al., 2003; Van Dyne and Pierce, 2004). Prior research states that psychological ownership emerges when certain human needs, such as a sense of belonging, self-identity, and efficacy, are satisfied (Pierce et al., 2003; Van Dyne and Pierce, 2004). By providing a setting for human survival and development, nature possesses the functional attributes to meet the requirement for space (Clayton and Opotow, 2003; Schultz and Tabanico, 2007). In addition to functional attributes, the greatest value of nature lies in its symbolic significance and its ability to strengthen self-identity through symbols (Schultz and Tabanico, 2007). Previous studies have shown that the natural environment may enable individuals to become perceptive knowers and establish positive values (Clayton and Opotow, 2003). Thus, we understand and position ourselves more effectively in the world. Meanwhile, during the process of the individual’s frequent interaction with the natural environment, the use of natural resources, physical and mental investment, and methods of coping with challenges do not only extend nature to self-cognitive representation and self-concept modification but also reflect their sense of self-realization efficacy, thereby inducing a psychological ownership of nature (Clayton and Opotow, 2003; Pierce et al., 2003; Van Dyne and Pierce, 2004; Schultz and Tabanico, 2007).

Psychological ownership is an individual’s sense of identity and connection to material and immaterial objects, including nature (Pierce et al., 2003; Van Dyne and Pierce, 2004). Factors that expand the self-concept to include nature and strengthen the connection between oneself and nature may influence psychological ownership; we suggest that awe is an example of a factor that achieves this effect. First, awe reinforces an individual’s focus on the natural world. Awe makes individuals feel self-diminished and insignificant, enabling them to overcome urges related to their daily needs and desires, encouraging them to pay more attention to others and the natural environment, and to focus on the sustainable development of nature (Keltner and Haidt, 2003; Piff et al., 2015; Jiang et al., 2018). Second, awe strengthens the connection between the individual and the natural world. Awe is a highly connected experience that makes people feel that they are no longer isolated individuals, and that they are closely connected to other people, objects, and the natural environment (Haidt, 2003; Pappas and Friedman, 2007). In such a state, they see themselves as part of a larger collective and feel oneness with the external world. Third, awe expands individuals’ self-concept. Broaden-and-build theory holds that awe endows people with a new perspective on their lives and enables them to see themselves and the world from a different angle (Fredrickson, 2001; Bonner and Friedman, 2011; Jiang et al., 2018). The more intense the awe experienced by individuals, the less likely they are to choose terms such as “special” or “unique” over terms like “individual” or “inhabitant of the earth” to define themselves, and they are more likely to emphasize their membership of a larger whole (Shiota et al., 2007). This process of extending one’s self to a larger entity, such as nature, can be expressed as natural-based psychological ownership – that is, the feeling that one is in possession of nature and natural resources.

Once psychological ownership has been established, a sense of ownership is activated, and feelings of responsibility and accountability for the target are strengthened (Dawkins et al., 2017). People who exhibit high psychological ownership of nature are motivated to engage in responsible behavior and protect, maintain, care for, and possibly even defend their possessions when necessary (Potdar et al., 2018). Prior research on psychological ownership in the context of natural resource management has found that psychological ownership reduces conflicts in natural resource exploitation and forms a cooperative relationship that is conducive to the sustainable development of the natural environment (Matilainen et al., 2017). In addition, endowment effect theory indicates that, aside from improving the individual’s sense of possession and emotional connection to the possession, psychological ownership increases the individual’s degree of sensitivity and pain in response to the loss of the possession, thus prompting the individual to take loss avoidance measures (Carmon and Ariely, 2000). Therefore, enhancement of an individual’s psychological ownership of nature leads to the perception that the loss of nature is equivalent to a loss of self (Kunchamboo et al., 2017); in this sense, altruistic environmental protection becomes a personal goal. A strong sense of psychological ownership inspires individuals to protect and attach more importance to nature (Kunchamboo et al., 2017) and, in turn, to adopt the practice of green consumption. Thus, we propose:

H2: The effect of awe on green consumption is mediated by nature-based psychological ownership.

## The Moderating Effect of Self-Construal

When the needs of belongingness, self-identity, and efficacy are satisfied, psychological ownership becomes affected by other factors, such as individual characteristics (Pierce et al., 2003; Dawkins et al., 2017). There are differences between individuals in terms of how they relate to others around them and their social environment, and such differences affect the degree of connection between individuals and their possession. In turn, these affect their psychological ownership. We define this individual difference as “self-construal,” referring to the collection of ideas, feelings, and actions that connect or distinguish one from others (Singelis, 1994). Self-concept theory divides self-construal into independent self-construal and interdependent self-construal (Singelis, 1994). Independent self-construal refers to the process of defining oneself based on perceptions about the wholeness and uniqueness of each person’s internal attributes that emphasizes independence (Wu et al., 2018). By contrast, interdependent self-construal refers to a self-definition based on one’s perceptions of not being separated from the social context but as one who is less differentiated and more connected to others, thereby emphasizing dependence and relevance (Wu et al., 2018).

Unlike independent self-construal individuals who attach importance to their own interests and self-goal realization, individuals with interdependent self-construal attach more importance to the interpersonal relationship between themselves and others, regard themselves as a member of the collective, and enact behavioral responses guided by collective interests and goals (Lee and Pounders, 2019). Interdependent self-construal individuals emphasize psychological ownership based on nature, and strive to interact with people, objects, and scenes in nature (Dawkins et al., 2017; Matilainen et al., 2017). The deepening of interaction solidifies and strengthens an individual’s identity and psychological connection to nature, and nature therefore gradually becomes the extension of self-concept construction, expression, maintenance, and even enhancement (Clayton and Opotow, 2003; Pierce et al., 2003; Van Dyne and Pierce, 2004; Kunchamboo et al., 2017). Over time, an interdependent self-construal individual in the natural environment no longer sees themselves as an isolated individual, but rather as a member of the larger collective or even the whole life collective. Therefore, compared with independent self-construal individuals, interdependent self-construal individuals are more likely to have a strong sense of psychological ownership, especially after experiencing awe.

H3: The awe experience increases people’s psychological ownership and subsequent green consumption upon the activation of interdependent self-construal; however, this mediation effect is attenuated if independent self-construal is activated.

## Methods and Results

### Experiment 1

In Experiment 1, we initially tested our hypothesis (i.e., awe promotes green consumption). One proxy was used to measure green consumption. In consumer psychology, green purchase behavior is the intention to purchase environmentally friendly or sustainable products in order to minimize the potentially negative environmental impact of purchases (Lee, 2008; Deepak and Rishi, 2018). Thus, by a narrative recall task, which has been well validated and widely used in prior research, we compared and determined whether the subjects experiencing awe (vs. happiness vs. neutral) were more willing to engage in green purchase behavior.

#### Participants and Procedure

All participants voluntarily participated and signed an informed consent form. The next steps met the requirements of the Ethics Committee of Wuhan University. The experiment used a three-cell (emotion: awe vs. happiness vs. neutral) between-subjects design. A total of 102 undergraduates (M_age_ = 22.68 years; 42 males) completed the study. The participants were randomly assigned to one of three scenarios and then asked to recall and describe a specific scenario to elicit the target emotions. The three scenarios were awe, happiness, and a neutral condition. The detailed instructions they were given are outlined below (adopted from Piff et al., 2015; Bai et al., 2017).

##### Awe condition

Awe arises when we encounter something immense in size, quantity, scope, complexity, ability, or social bearing, such that it transcends our current understanding of the world, environment, or self. Please think about a recent natural scene that caused you to feel awe. This scene might have been a spectacular sunset, a majestic mountain, a towering tree, a magnificent cloud, or any natural scenery that surprised you.

##### Happiness condition

When your inner needs and desires are satisfied, you experience happiness. Please think about a recent scene that made you feel happy. This might have been a birthday party, happy family gathering, good time with friends, or other moment that made you feel good.

##### Neutral condition

Please think about recent daily life activities, such as riding a bicycle, studying in the classroom, etc.

Subsequently, participants completed a survey using a green purchase behavior scale (1 = strongly disagree, 7 = strongly agree; α = 0.809) (Lee, 2008). One sample item was “I prefer green products over non-green products when their product qualities are similar.” They were then asked to report their current emotional state using single items (1 = not at all, 7 = extremely): anger, awe, disgust, fear, pride, sadness, and happiness (Piff et al., 2015). Each participant received a small gift as a token of appreciation.

#### Results and Discussion

##### Manipulation checks

The results from a multivariate analysis of variance (MANOVA) (Table 1) showed that the awe condition produced higher levels of awe than the happiness and neutral conditions did. In comparison, the happiness condition yielded higher levels of happiness compared to the awe and neutral conditions. Furthermore, the awe group felt happier than the neutral group and prouder than the other two groups. Other emotions – namely, anger, disgust, fear, and sadness – did not significantly differ among the three groups. These findings suggest that the target emotion manipulation was successful.

**TABLE 1:** Mean scores for self-reported emotional states in Experiment 1, 2, 3, and 4 (SDs in Parentheses).

	**Experiment 1**			**Experiment 2**			**Experiment 3**		
	**G1**	**G2**	**G3**	**G1**	**G2**	**G3**	**G1**	**G2**	**G3**
	
	**Awe**	**Happiness**	**Neutral**	**Awe**	**Nature**	**Neutral**	**Non-nature**	**Negative**	**Neutral**
Anger	1.05 (0.23)	1.06 (0.24)	1.10 (0.31)	1.11 (0.32)	1.14 (0.35)	1.13 (0.33)	1.34 (0.60)	1.35 (0.53)	1.27 (0.72)
Awe	5.71(0.84)^b,c^	1.44(0.79)^a^	1.60(1.04)^a^	5.69(0.95)^b,c^	1.50 (0.71)	1.43 (0.87)	5.66(0.92)^c^	5.59(0.88)^c^	1.49(0.89)^a,b^
Disgust	1.11 (0.31)	1.06 (0.24)	1.10 (0.31)	1.07 (0.25)	1.05 (0.22)	1.08 (0.27)	1.34 (0.84)	1.30 (0.51)	1.29 (0.69)
Fear	1.26 (0.45)	1.24 (0.74)	1.23 (0.63)	1.29 (0.51)	1.19 (0.51)	1.20 (0.52)	1.36(0.79)^b^	3.52(0.81)^a,c^	1.33(0.77)^b^
Pride	2.82(1.14)^b,c^	2.09(0.93)^a^	1.67(0.92)^a^	3.69(0.60)^b,c^	1.21(0.52)^a^	1.30(0.69)^a^	3.62(0.71)^b,c^	1.33(0.63)^a^	1.38(0.75)^a^
Sadness	1.37 (0.67)	1.21 (0.59)	1.37 (0.72)	1.29 (0.59)	1.21 (0.47)	1.23 (0.48)	1.51(0.98)^b^	3.41(0.72)^a,c^	1.44(0.87)^b^
Happiness	2.84(0.64)^b,c^	5.97(0.87)^a,c^	1.20(2.06)^a,b^	3.07(0.89)^b,c^	1.45(0.67)^a^	1.43(0.55)^a^	3.72(0.62)^b,c^	1.26(0.61)^a^	1.27(0.62)^a^

*For Experiment 1, 2, and 3, ^*a*^The means are different from those in group 1 (G1) (*p* < 0.05); ^*b*^The means are different from those in group 2 (G2) (*p* < 0.05); ^*c*^The means are different from those in group 3 (G3) (*p* < 0.05).*

##### Main effect analysis

One-way ANOVA results showed that there were significant differences in green purchase behavior among the three groups, *F*(2,99) = 11.33, *p* = 0.000, $\eta_{p}^{2}=0.19$. *Post hoc* analysis revealed that participants in the awe condition (M_awe_ = 5.47, SD = 0.68) were more likely to engage in green purchase behavior than those in the happiness (M_happiness_ = 4.92, SD = 0.68) and neutral conditions (M_neutral_ = 4.76, SD = 0.60; awe vs. happiness: 95% confidence interval (CI) for mean difference [0.18, 0.93], *p* = 0.002; awe vs. neutral: 95% CI for mean difference [0.33, 1.10], *p* = 0.000). Meanwhile, there was no significant difference between the happiness and neutral groups (95% CI for mean difference [−0.23, 0.55], *p* > 0.050). These findings prove our prediction that awe experiences promote green purchase behavior, whereas happiness and neutral experiences do not. Thus, the results support H1.

##### Discussion

Experiment 1 preliminarily proves, through a narrative recall task, that an awe experience promotes green purchase behavior. Notably, the ability of awe to promote green purchase behavior was not simply due to positive valence because participants who were induced to feel the positive emotion of happiness were less likely to engage in green purchase behavior compared to those induced to feel awe. The findings of Experiment 1 also indicate that such an effect was due to the fact that awe promoted green purchase behavior, thereby suggesting that participants in the awe condition were more likely to engage in green purchase behavior than those in the neutral condition were. Although Experiment 1 initially achieved the desired effect, the narrative recall method highlighted the participants’ retrospective self-report on specific things, rather than the awe experience itself, which may have had some influence on their subsequent behavioral choice. In order to eliminate this effect, Experiment 2 was conducted to further verify the results of Experiment 1.

### Experiment 2

Experiment 2 was first conducted to examine whether the causal relationship between awe and green purchase behavior was mediated by psychological ownership. Second, it aimed to identify ordinary natural scenes (e.g., grass) that would not qualify as belonging to the category of natural scenes that inspire awe experiences (i.e., appreciation of majestic and extraordinary scenery) (Joye and Bolderdijk, 2015; Zhao et al., 2018). We added ordinary natural scenes as a control group to eliminate its interference.

#### Participants and Procedure

This experiment used a three-cell (emotion: awe vs. nature vs. neutral) between-subjects design. A total of 127 undergraduates (M_age_ = 23.46 years; 67 males) completed the study. All participants were randomly assigned to watch a series of four slideshows of known elicitors of awe (e.g., sunsets, mountain peaks, waterfalls, and towering tree), nature (e.g., leaves, grass, cactus, and sapling), or neutral (e.g., desk, bench, cup, and micro USB), adopted from Piff et al. (2015) and Zhao et al. (2018). After watching the slideshows, participants answered a set of questions using a seven-point Likert scale to measure their green purchase behavior, as in Experiment 1 (α = 0.809; Lee, 2008), and their psychological ownership (α = 0.843; Van Dyne and Pierce, 2004). One sample item for psychological ownership was “I sense that nature is ours.” They were also asked to fill out a self-diminishment scale (i.e., feeling that one’s own being or goals are less significant; Rudd et al., 2018). Given that self-diminishment influences the relationship between awe and pro-sociality (Piff et al., 2015), we used it as a control variable to clarify the mediating effect of psychological ownership. To check the manipulation, participants also completed questions to assess their current emotional state, as in Experiment 1 (1 = not at all, 7 = extremely) (Piff et al., 2015).

#### Results and Discussion

##### Manipulation checks

We conducted a multivariate analysis of variance (MANOVA) on the emotion items. The results (Table 1) showed that the awe condition produced a higher level of awe than the nature and neutral conditions. Furthermore, the awe group also felt happier and prouder than the two other groups. Other emotions, namely, anger, disgust, fear, and sadness, were not significantly different among the three groups. These findings suggest that the target emotion manipulation is successful.

##### Main effect analysis

One-way ANOVA results showed that there were significant differences in green purchase behavior among the three groups, *F*(2,124) = 10.43, *p* = 0.000, $\eta_{p}^{2}=0.14$. *Post hoc* analysis revealed that participants in the awe condition (M_awe_ = 5.75, SD = 0.79) were more likely to engage in green purchase behavior than those in the nature (M_*natural*_ = 5.11, SD = 0.71) and neutral conditions (M_neutral_ = 5.04, SD = 0.91; awe vs. nature: 95% CI for mean difference [0.23, 1.05], *p* = 0.001; awe vs. neutral: 95% CI for mean difference [0.30, 1.13], *p* = 0.000). Meanwhile, there was no significant difference between the nature and neutral groups (95% CI for mean difference [−0.35, 0.49], *p* > 0.050). As expected, awe experiences promoted green purchase behavior, whereas happiness and neutral experiences did not. Thus, our results support H1.

##### Mediating effect analysis

**FIGURE 1:**
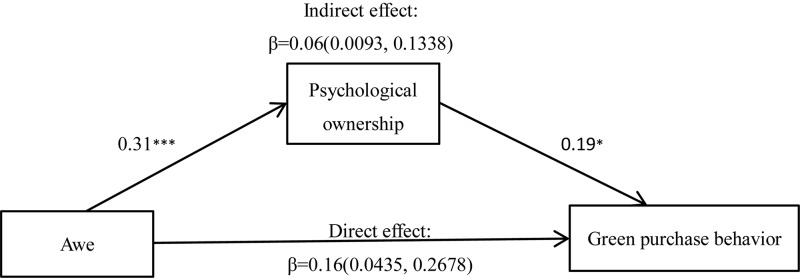
Mediation analysis of psychological ownership. ^∗^*p* < 0.050 and ^∗∗∗^*p* < 0.001.

Regression analysis showed that psychological ownership was positively related to green purchase behavior, *r* = 0.38, *p* = 0.000. Therefore, a mediation analysis was conducted to test whether the awe induction increased the participants’ willingness to engage in green purchase behavior by increased psychological ownership. We tested the proposed mediating effect using a bootstrapping procedure for mediator models (Model 4; *N* = 5000; Hayes, 2013), as shown in Figure 1. After controlling self-diminishment, we found that 95% CI for the indirect effect did not contain 0, suggesting that psychological ownership plays a mediating effect between awe and green purchase behavior.

##### Discussion

Different emotional manipulation modes from those used in Experiment 1 were applied to revalidate H1 and thereby indicate the stability of the experimental results. Consequently, excluding the influence of ordinary natural scenes, Experiment 2 again proved that, of the three conditions, only awe promoted green purchase behavior. Furthermore, bootstrap results showed that the influence of awe on green purchase behavior was generated through psychological ownership, thus proving that psychological ownership played a mediating effect, and thus supporting H2.

### Experiment 3

Awe is often triggered by spectacular natural landscapes; however, it can also be triggered by various natural disasters, such as volcanoes and tornadoes, as well as by extraordinary social elicitors, such as the Egyptian pyramids and the Great Wall of China, both of which have been successfully used in previous studies (Keltner and Haidt, 2003; Piff et al., 2015). Therefore, Experiment 3 was conducted to introduce two variants of awe inductions (negative and non-nature induction of awe), from which we were able to examine the generalizability of our effects.

#### Participants and Procedure

The experiment used a three-cell (emotion: non-nature awe vs. negative awe vs. neutral) between-subjects design. A total of 138 undergraduates (M_age_ = 23.46 years; 67 males) completed the study. All participants were randomly assigned to watch a series of four slideshows of known elicitors of non-nature awe (e.g., Egyptian pyramids, the Great Wall of China, Gandhi, and Da Vinci’s paintings, such as *Mona Lisa*), negative awe (e.g., tornados, lightning strikes, tsunamis, and volcanoes), or neutral (e.g., desk, bench, cup, and bedroom), adopted from Piff et al. (2015) and Zhao et al. (2018). They were then asked to fill out the green purchase behavior (α = 0.806), psychological ownership (α = 0.832), and control variable self-diminishment scales as described in Experiment 2. Manipulation checks were also conducted to assess the participants’ current emotional state.

#### Results and Discussion

##### Manipulation checks

The MANOVA results on the emotion items in Table 1 showed that non-nature awe and negative awe conditions produced higher levels of awe than the neutral condition, while no significant difference in awe experience between the non-natural and negative awe groups existed. Furthermore, the non-natural awe group also felt happier and prouder than the two other groups. In comparison, the negative awe group felt greater fear than the two other groups. The ratings of other emotions were not significantly different among the three groups. These findings suggest that the target emotion manipulation is successful.

##### Main effect analysis

Replicating the Experiment 2 analysis, this testing showed that there were significant differences in green purchase behavior among the three groups, *F*(2,135) = 14.66, *p* = 0.000, $\eta_{p}^{2}=0.18$. *Post hoc* analysis revealed that, compared with the neutral group (M_neutral_ = 4.94, SD = 0.78), the non-natural and negative awe groups were more willing to engage in green purchase behavior (M_*non–nature*_ = 5.71, SD = 0.70; M_*negative*_ = 5.61, SD = 0.73; non-natural awe vs. neutral: 95% CI for mean difference [0.40, 1.13], *p* = 0.000; negative awe vs. neutral: 95% CI for mean difference [0.30, 1.04], *p* = 0.000). In comparison, no significant difference can be found between the non-natural and negative awe groups (non-natural awe vs. negative awe: 95% CI for mean difference [−0.26, 0.46], *p* > 0.050). As expected, non-natural awe and negative awe promotes green purchase behavior, thereby proving H1 once more.

##### Mediating effect analysis

**FIGURE 2:**
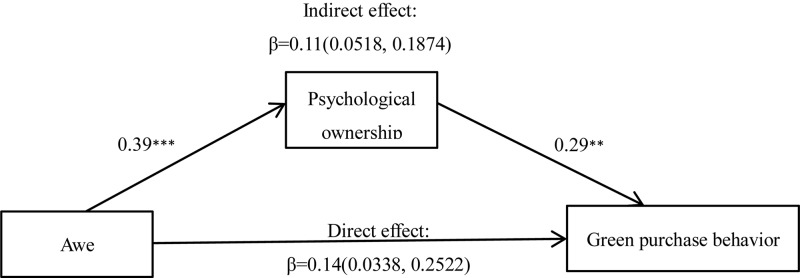
Mediation analysis of Experiment 3. ^∗^*p* < 0.010 and ^∗∗∗^*p* < 0.001.

Regression analysis showed that psychological ownership was positively related to green purchase behavior, *r* = 0.46, *p* = 0.000. Therefore, a mediation analysis was conducted to examine whether the awe conditions influenced participants’ green purchase behavior via psychological ownership. We tested the proposed mediating effect using a bootstrapping procedure for mediator models (Model 4; *N* = 5000; Hayes, 2013), as shown in Figure 2. After controlling self-diminishment, we found 95% CI for the indirect effect did not contain 0, meaning the mediating effect of psychological ownership is supported.

##### Discussion

Experiment 3 compared the awe experience triggered by different stimuli and found that non-natural awe and negative awe alike can both promote green purchase behavior, indicating that the influence of awe on green purchase behavior is not limited to the typical awe experience. In addition, Experiment 3 once again demonstrated the mediating effect of psychological ownership through the test of different awe experiences.

### Experiment 4

The fourth experiment employed a 2 (emotion: awe vs. neutral) × 2 (self-construal: independent vs. interdependent) between-subjects design to test H3 – i.e., to verify the moderating effects of self-construal. To improve the externality of the experiment, 222 Master of Business Administration students (M_age_ = 29.88 years; 92 males) were chosen to take part.

#### Procedure

Participants were asked to read a story about a city trip, circle the appropriate personal pronouns, and count how many pronouns they had circled. The pronoun circle method enabled the participants to pay more attention to the situation, and effectively activate their independent and interdependent selves at a given time. This method has been widely used in previous studies to manipulate self-construal (Hong and Chang, 2015; Li and Fan, 2018). For this experiment, personal pronouns such as “we” and “our” were used in the context of interdependent self-construal. In addition, personal pronouns such as “I” and “my” were used in independent self-construal to describe the story. Furthermore, the participants filled out a two-item focus scale for a self-construal manipulation test (e.g., “The above travel notes made me think more about myself” and “The above travel notes made me think more about my family and friends”; 1 = strongly disagree, 7 = strongly agree; Hong and Chang, 2015). Participants were then made to watch four slideshows of natural landscapes that inspire awe, as in Experiment 2. Afterward, they filled out green purchase behavior (α = 0.852), psychological ownership (α = 0.758), and self-diminishment scales, and reported their current emotional state as described in Experiment 2.

#### Results and Discussion

##### Manipulation checks

Manipulations were tested in a series of *t*-tests. As expected, the awe group experienced higher levels of awe (M_awe_ = 5.86, SD = 0.95, M_neutral_ = 1.39, SD = 0.84, *t*(220) = 37.36, *p* = 0.000), pride (M_awe_ = 3.18, SD = 1.01, M_neutral_ = 1.48, SD = 0.82, *t*(220) = 13.81, *p* = 0.000), and happiness (M_awe_ = 3.07, SD = 1.02, M_neutral_ = 1.96, SD = 0.96, *t*(220) = 8.31, *p* = 0.000) than the neutral group. The manipulation did not have a significant effect on anger, disgust, fear, sadness (*t*s < 0.94, n.s.). For self-construal, participants with independent self-construal focused significantly more on themselves (M_*self*_ = 5.85, SD = 0.92, M_*other*_ = 2.10, SD = 0.97, *t*(109) = 24.21, *p* = 0.000), whereas those with interdependent self-construal focused significantly more on others (M_*other*_ = 5.25, SD = 1.03, M_*self*_ = 2.42, SD = 1.03, *t*(111) = 19.07, *p* = 0.000).

##### Main effect analysis

ANOVA was conducted to test the moderating effect. Results showed that awe had a significant direct effect on green purchase behavior, *F*(1,218) = 5.21, *p* < 0.050,$\eta_{p}^{2}=0.023$, and that the awe group was more willing to engage in green buying behavior than the neutral group (M_awe_ = 5.56, SD = 0.67, M_neutral_ = 5.34, SD = 0.74). In addition, the interactive effects of awe and self-construal on green buying behavior were significant, *F*(1,218) = 5.90, *p* < 0.050,$\eta_{p}^{2}=0.026$. The simple effect analysis results showed that when the interdependence self-construal was activated, the awe group participants were more willing to engage in green purchase behavior (M_awe_ = 5.79, SD = 0.09, M_neutral_ = 5.36, SD = 0.10), *F*(1,218) = 11.13, *p* = 0.001, $\eta_{p}^{2}=0.049$. However, the difference between the two groups was not significant if the independent self-construal was activated (M_awe_ = 5.31, SD = 0.09, M_neutral_ = 5.32, SD = 0.09), *F*(1,218) = 0.011, *p* > 0.050.

##### Mediating effect analysis

Results of the ANOVA test showed the significant interaction effects of awe and self-construal on psychological ownership, *F*(1,218) = 9.39, *p* = 0.002, $\eta_{p}^{2}=0.041$. The simple effect analysis results indicated that when the interdependence self-construal was activated, the awe group (vs. the neutral group) participants had a stronger sense of psychological ownership (M_awe_ = 5.71, SD = 0.62, M_neutral_ = 5.19, SD = 0.68), *F*(1,218) = 15.98, *p* = 0.000, $\eta_{p}^{2}=0.068$. However, the effect of awe on psychological ownership was not significant if the independent self-construal was activated (M_awe_ = 5.02, SD = 0.69, M_neutral_ = 5.07, SD = 0.79), *F*(1,218) = 0.117, *p* > 0.050.

**FIGURE 3:**
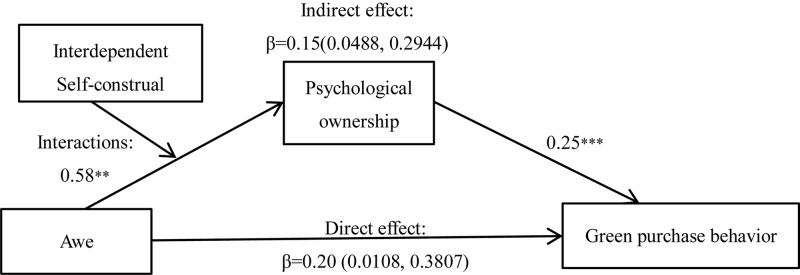
Analysis of self-construal as moderator. ^∗^*p* < 0.010 and ^∗∗∗^*p* < 0.001.

Figure 3 reveals the results of the mediation analysis (Model 7; *N* = 5000; Hayes, 2013). After controlling self-diminishment, findings showed that the mediating effect of psychological ownership between awe and green purchase behavior (95% CI: [0.0590, 0.2500]), β = 0.1351 was significant only when interdependent self-construal was activated, whereas the mediating effect of psychological ownership was not significant if the independent self-construal was activated (95%CI: [−0.0933, 0.0590]). The significant indirect effect of self-construal indicated that psychological ownership mediates the effect of awe on green purchase behavior but only when interdependent self-construal was activated.

##### Discussion

The results of Experiment 4 are consistent with those of Experiments 1–3, thereby proving the positive effect of awe on green purchase behavior (H1) and the mediating effect of psychological ownership (H2). Notably, Experiment 4 demonstrated that self-construal, especially interdependent self-construal, is central to the enhanced mediating effect of psychological ownership (H3).

## Discussion and Conclusion

Numerous scenes inspire awe experiences in daily life, including majestic natural landscapes, elegant art, and outstanding contributions of advanced individuals. Awe experiences also often occur in consumption scenarios, such as Apple’s flagship stores, Samsung advertising, and tourist resorts (Rudd et al., 2018). In the realm of academic research, Piff et al. (2015) found that the awe experience promotes pro-social behaviors among individuals. Nevertheless, research on the downstream effect of awe experience is still in the primary stage (Jiang et al., 2018; Rudd et al., 2018). Guided by the conviction that lucid waters and lush mountains are invaluable assets, we wanted to assess whether the awe often induced by natural landscapes is conducive to the practice of green development in the field of consumption. Thus, the present study investigated the possibility of stimulating green consumption – a consumption mode that best embodies the concept of green development – via awe experiences.

First, through targeted emotion manipulation tests using a narrative recall task (Experiment 1) or slideshows (Experiments 2–4), we found that awe was the only emotion, out of the general positive and neutral emotions incited in participants, to promote green consumption behaviors, which was consistent with Piff et al. (2015) finding that awe promotes pro-social behavior. Experiment 2 compared ordinary natural scenes with extraordinary nature scenes and excluded the interference of ordinary natural landscapes.

Second, Experiment 2 investigated the mediating effect of psychological ownership. As a positive social-functional emotion, awe broadens the self-concept, emphasize the sense of connection between the self and others and nature, and enhance one’s view of oneself as part of a larger entity (Fredrickson, 2001; Shiota et al., 2007; Bonner and Friedman, 2011; Jiang et al., 2018). Bethelmy and Corraliza (2019) stated that awe brings transcendental changes to individuals psychologically. The generation of psychological ownership based on nature stems from the psychological connection and integration between nature and the self, which makes people feel that nature belongs to them or to the collective as a whole (Pierce et al., 2003; Van Dyne and Pierce, 2004). Nature-based psychological ownership results from self-concept extension and cognitive transformation. Thus, there is reason to believe that awe enhances psychological ownership based on nature. Moreover, the stronger the sense of psychological ownership someone exhibits, the greater their motivation is to engage with responsible behavior (Potdar et al., 2018), such as green consumption. Consistent with these concepts, we predicted and demonstrated that individuals with a stronger awe experience have a stronger sense of psychological ownership and are therefore more likely to engage in green consumption (H2). Expanded testing by introducing non-natural awe and negative awe in Experiment 3 proved that both negative awe and non-natural awe strengthen psychological ownership and promote green consumption (H2).

Third, through the pronoun-identification method that was employed to conduct a manipulation test of self-construal, we further verified a boundary condition of the effect of awe on green consumption. Awe shifts self-representation away from individual traits and preferences to more collective traits (Bai et al., 2017). Furthermore, interdependent self-construal is oriented to others or the collective (Lee and Pounders, 2019). Therefore, when such construal is activated, it leads to individual’s stronger psychological ownership via the enhancement of the awe experience, leading to the greater possibility of them engaging in green consumption. However, this process is absent when independent self-construal is activated.

### Implications

This paper contributes to previous research and practice through three main aspects. First, most previous studies on the antecedents of green consumption were conducted from a constraint perspective, thereby ignoring the potential impact of autonomous choice on green consumption. Autonomy is a fundamental psychological need of human beings (Kouchaki et al., 2018). Numerous studies have shown that people tend to have a strong motivation to pursue the right to make autonomous choices, while moral ethics may limit their ability to perceive choices (Kouchaki et al., 2018). As a typical mode of ethical consumption based on self-interest sacrifice, green consumption is sometimes performed under social norms or impression management (Auger and Devinney, 2007; Gleim et al., 2013). This is a decision that should be made deliberately, rather than as a result of free choice (Kouchaki et al., 2018), thus limiting the autonomy of choice to a certain extent. Behavioral driver studies have found that emotion is one of the most important drivers and should not be ignored; in fact, emotion may even surpass cognition and play a leading role (Bagozzi et al., 1999). Compared with negative emotions, positive emotions result from the satisfaction of individual autonomy and contribute to pro-social behavior (Bagozzi et al., 1999; Kouchaki et al., 2018). Awe, a positive emotion of self-transcendence, is considerably helpful in stimulating the intrinsic motivation of environmental protection (Bethelmy and Corraliza, 2019) and improving the possibility of green consumption. Compared with macro-government intervention and moral constraints, awe offers a way to enhance environmental protection that is effective, considerably low in cost, and easy to manipulate and realize. Green consumption should be encouraged in future management practices through outstanding environmental protection activities, ritual activities, internal corporate environment, and relevant advertisements that elicit awe.

Second, the mechanism of psychological ownership aids in advancing the subjective initiative of individual engagement in green consumption. Green consumption is oriented toward the welfare and sustainable development of all humans, and its positive effects are the results of collective, long-term, and continuous efforts (Lee, 2008; Griskevicius et al., 2010; Gleim et al., 2013; Karmarkar and Bollinger, 2015). Faced with such ecological and environmental protection measures that require broader, national-level action, the individual’s sense of citizenship and responsibility is diluted, which leads to the dilemma of the “bystander effect” (Gleim et al., 2013; Shaw et al., 2016). However, if the idea that “the Earth is our homeland” can be aroused in individuals, and their psychological ownership of nature enhanced, individuals’ sense of responsibility to protect the environment improves. Awe is a high connectivity experience that helps to increase individuals’ connection to larger entities, including nature, which is the main path of psychological ownership. In addition, awe, to a certain extent, reflects a high recognition and respect nature, weakens the anthropocentric development view, improves perceptions of the dominant position of nature, and facilitates a harmonious and equal development relationship between human beings and nature (Thompson and Barton, 1994; Cheung et al., 2014; Stellar et al., 2017). Hence, awe reduces the sense of individual ownership of nature. Future managers should thus promote the idea that “the Earth is our homeland” through slogans, promotional videos, and other means. In this way, managers fundamentally shape the sense of ownership and enhance individuals’ sense of responsibility and mission to protect the environment.

Finally, our study enriches the extant green consumption literature by introducing self-construal into the analysis. Most existing advocacy of green consumption has been based on the same self-definition level, ignoring the differentiation effect of self-definition (e.g., Sheth et al., 2011; Karmarkar and Bollinger, 2015). In fact, compared with independent self-construal, interdependent self-construal individuals are more inclined to conduct behavioral responses guided by others and collective interests (Lee and Pounders, 2019), and they are more likely to participate in green consumption – a collective activity that requires the participation of society as a whole. In future management activities, especially in advertising, more personal pronouns (e.g., “we” and “our”) should be used in language expression, and more pictures should be presented that depict families and groups.

### Limitations

Although our data provide converging evidence for the proposed theoretical framework, this work is not without limitations, which warrant future research. First, although we strictly manipulated the experimental research, all research was conducted in the laboratory, which, to some extent, limited their externality. Given that individuals visiting tourist resorts or other magnificent natural landscapes are more likely to experience awe (Keltner and Haidt, 2003; Piff et al., 2015; Bethelmy and Corraliza, 2019), in future studies field experiments in tourist resorts can be considered to further enhance the externality and robustness of the research. Second, green consumption in this study was mostly measured via survey questionnaires. However, with respect to ethical consumption, the survey results may have been affected by the social desirability effect (Auger and Devinney, 2007; Gleim et al., 2013). Thus, future research should be conducted in real consumption scenarios to minimize experimental deviation caused by the “inconsistency between words and deeds” in ethical consumption.

## Data Availability Statement

The datasets generated for this study are available on request to the corresponding author.

## Ethics Statement

All procedures performed in studies involving human participants were in accordance with the 1964 Helsinki Declaration and its later amendments or comparable ethical standards. All subjects gave written informed consent in accordance with the Declaration of Helsinki. The protocol was approved by the Ethics Committee of the Department of Marketing and Tourism Management, Wuhan University.

## Author Contributions

LW involved in all steps of the study. LW, PS, XL, and FS conducted the experiments. LW and PS performed the statistical analysis. LW wrote the first draft of the manuscript. LW, GZ, and XL revised and read the manuscript. GZ provided the funding support. All authors read and approved the submitted version.

## Conflict of Interest

The authors declare that the research was conducted in the absence of any commercial or financial relationships that could be construed as a potential conflict of interest.
